# Advances in COVID-19 and Cancer Research

**DOI:** 10.3390/jcm13144143

**Published:** 2024-07-16

**Authors:** Laura Franza, Rossella Cianci

**Affiliations:** 1 Fondazione Policlinico Universitario A. Gemelli, Istituto di Ricerca e Cura a Carattere Scientifico (IRCCS), 00168 Rome, Italy; rossella.cianci@unicatt.it; 2 Emergency Department, Azienda Ospedaliero-Universitaria di Modena, Largo del Pozzo, 71, 41125 Modena, Italy; 3 Department of Translational Medicine and Surgery, Catholic University of Sacred Heart, 00168 Rome, Italy

The global health crisis caused by COVID-19 has radically changed the management of several diseases. In this Topic, we explore the advances made in the field of the prevention, diagnosis, and treatment of COVID-19 and its impact on cancer care [1].

Overall, the new domain that emerged during the pandemic was the development and use of mRNA vaccines. This new type of vaccine can “train” the production of antibodies against specific antigens normally produced by different pathogens or neoplastic cells [2]; therefore, it has the potential to aid in therapy against different types of cancer [3]. However, as with any medical procedure, vaccinations can present some side effects and can lead to hypersensitivity reactions. It is generally agreed that the benefits outweigh the risks, but during the vaccination campaign against COVID-19, hypersensitivity reactions were among the various scare-mongering tactics used by anti-vaxxers to question the vaccination process [4]. As reported by Kurniawan et al., not only did the vaccines cause generally mild hypersensitivity reactions but it was also possible to predict whether or not people would experience such reactions through provocative tests (Contribution 1). Vaccines, overall, changed the trajectory of the COVID-19 pandemic, and their use impacted the transition from it being epidemic to endemic (Contribution 2). Subsequently, the progress that has been made in designing mRNA vaccines is being applied to design cancer vaccines, which has the potential to create a significant impact on patients’ lives.

During the different stages of the pandemic, different therapeutic strategies were tested, with varying degrees of success [5]. The use of convalescent plasma has proven to be effective in patients with mild-to-severe pneumonia, likely through the modulation of inflammatory cytokines such as IFN-β, IL-6, IL-10, and IFN-α (Contribution 3). The levels of these cytokines can be used to predict the likelihood of different outcomes of COVID-19, but even more precise predictors have been identified. Kattner et al., for instance, identified Krebs von den Lungen-6 (KL-6) as an accurate outcome predictor for COVID-19 pneumonia (Contribution 4), and Zhang et al. suggested that the dynamic monitoring of von Willebrand Factor and ADAMTS13 can predict mortality in hospitalized patients (Contribution 5). Some populations have presented particular vulnerabilities to SARS-CoV-2 infection, such as children and pregnant women. The neutrophil-to-lymphocyte ratio (NLR) has been shown to have a high level of accuracy in predicting negative outcomes in pregnant women (Contribution 6). In children, the development of multisystem inflammatory syndrome is of particular concern; consequently, Subramanian et al. analyzed data to evaluate whether it was possible to stratify patients based on the risk of developing more severe forms of COVID-19 (Contribution 7). The use of artificial intelligence in combining data has been fundamental in the creation of algorithms that help predict outcomes in people with COVID-19, and these could be utilized in different contexts, such as predicting outcomes and responsiveness to therapies in people battling cancer. Another population that is at a particularly high risk includes patients with malignancies [6]. As discussed by Na et al., malignancy in itself was a predictor of negative outcomes after SARS-CoV-2 infection, but it is worth noting that physical therapy could benefit this particular group (Contribution 8). Another potentially useful strategy in this group of patients and other more at-risk groups is the use of extracorporeal membrane oxygenation (ECMO). While data on its practice has not always been consistent, it was observed that, in particularly fragile populations, it could potentially change the course of the disease, mainly in its early stages (Contribution 9).

The diagnosis of COVID-19 was also a subject of research and advancement. The majority of testing for COVID-19 took place through the use of swabs to perform molecular tests, such as polymerase chain reaction (PCR) and other nucleic acid amplification tests (NAATs), or by detecting antigens from the virus [7,8]. The use of swabs offered many advantages, but it did not offer total accuracy; it was uncomfortable for those undergoing it and exposed operators to an increased risk of infection. A different approach to this issue has been proposed by Zhunissova et al., who suggested employing a combination of data to accurately diagnose SARS-CoV-2 infection (Contribution 10). Another aspect that needs to be taken into consideration is that PCR-based direct DNA sequencing methods require time and resources. Kyung et al. described a fluorometric method for the accurate detection of one missense mutation that changes leucine to arginine (L452R) in SARS-CoV-2 genomic RNA, and this method appears to have both high sensitivity and accuracy scores (Contribution 11).

Overall, the advances that have been made during the COVID-19 pandemic have dramatically changed the face of medicine. This pandemic has represented a new way of conceiving medicine, which is required in order to be able to obtain faster techniques for achieving precise diagnosis and appropriate therapies and face the new challenges that arise from the consequences of climate change [9]. Furthermore, the new development and use of mRNA vaccines signify a great opportunity for novel methods to be used in the long battle against cancer.
